# Inhibition of Endothelial Activation: A New Way to Treat Cerebral Malaria?

**DOI:** 10.1371/journal.pmed.0020245

**Published:** 2005-08-23

**Authors:** Samuel C Wassmer, George J Cianciolo, Valéry Combes, Georges E Grau

**Affiliations:** 1CNRS UMR 6020, Immunopathology Group, IFR 48, Faculty of Medicine, Université de la Méditerranée, Marseille, France,; **2**Department of Pathology, Duke University Medical Center, Durham, North Carolina, United States of America; Mahidol UniversityThailand

## Abstract

**Background:**

Malaria is still a major public health problem, partly because the pathogenesis of its major complication, cerebral malaria (CM), remains incompletely understood. However tumor necrosis factor (TNF) is thought to play a key role in the development of this neurological syndrome, as well as lymphotoxin α (LT).

**Methods and Findings:**

Using an in vitro model of CM based on human brain–derived endothelial cells (HBEC-5i), we demonstrate the anti-inflammatory effect of LMP-420, a 2-NH_2_-6-Cl-9-[(5-dihydroxyboryl)-pentyl] purine that is a transcriptional inhibitor of TNF. When added before or concomitantly to TNF, LMP-420 inhibits endothelial cell (EC) activation, i.e., the up-regulation of both ICAM-1 and VCAM-1 on HBEC-5i surfaces. Subsequently, LMP-420 abolishes the cytoadherence of ICAM-1-specific *Plasmodium falciparum*–parasitized red blood cells on these EC. Identical but weaker effects are observed when LMP-420 is added with LT. LMP-420 also causes a dramatic reduction of HBEC-5i vesiculation induced by TNF or LT stimulation, as assessed by microparticle release.

**Conclusion:**

These data provide evidence for a strong in vitro anti-inflammatory effect of LMP-420 and suggest that targeting host cell pathogenic mechanisms might provide a new therapeutic approach to improving the outcome of CM patients.

## Abstract 2

Targeting the inflammatory pathway with a novel small molecule inhibitor, LMP-420, might provide a new therapeutic approach to cerebral malaria.

## Introduction

Malaria results in up to 2.5 million deaths annually, with young children and pregnant women at greatest risk. *Plasmodium falciparum* causes the most severe and life-threatening form of malaria in man, cerebral malaria (CM). This complex cerebral syndrome includes several features in pediatric patients, notably the sequestration of parasitized red blood cells (PRBC) [1,2], platelets and leucocytes [3] in brain capillaries and post-capillary venules, systemic endothelial activation [4], and, as recently described, increased numbers of circulating endothelial microparticles (MP) [5].

Numerous studies provide evidence for the key role of tumor necrosis factor (TNF) in the pathogenesis of CM, and a clear relationship has been established between plasma concentrations of TNF levels and cerebral pathology [6–8]. In experimental CM, TNF-beta, now called lymphotoxin α (LT), was recently shown to be the principal mediator of pathogenesis [9]. Indeed, LT and TNF belong to the same family, interact with a common receptor, and could act together during the pathogenesis [10]. Both cytokines can activate endothelium and be responsible for an increase of MP release by human brain endothelium (S. C. Wassmer, V. Combes, F. Candal, I. Juhan-Vague, and G. E. Grau, unpublished data) [11].

In this report we test the anti-inflammatory activity of a newly designed 2-NH_2_-6-Cl-9-[(5-dihydroxyboryl)-pentyl] purine, named LMP-420. LMP-420 inhibits transcription of mRNA for TNF in a variety of human cell types including monocytes, T lymphocytes, neutrophils, adipocytes, and endothelial cells (ECs), and has a 50% inhibitory concentration (IC_50_) of 50 nM in human peripheral blood mononuclear cells (S. Haraguchi, N. K. Day, W. Kamchaisatian, M. Engele, S. Stenger, N. Tangsinmankong, J. W. Sleasman, S. V. Pizzo, and G. J. Cianciolo, unpublished data). In this study, using an in vitro co-culture model composed of human brain microvascular EC (HBEC-5i), and FCR-3 or FCR-3–derived *P. falciparum* strains, we aimed to assess the ability of LMP-420 to inhibit in vitro TNF and/or LT effects on brain endothelium, with particular attention to its activation, adhesiveness for malarial parasites, and vesiculation.

## Methods

### Reagents

LMP-420 (2-NH_2_-6-Cl-9-[(5-dihydroxyboryl)-pentyl] purine) was provided as a gift from LeukoMed, Inc. of Raleigh, North Carolina, United States. It was stored either as a dry powder under desiccation at −20 °C or at −20 °C as aliquots of a 10 mM stock solution in DMSO (tissue culture grade dimethylsulfoxide; Sigma, St. Louis, Missouri, United States).

### Human Brain Endothelial Cells (HBEC-5i)

Purified human brain microvascular EC (HBEC-5i [12]) were seeded on culture flasks and grown to confluence in DME/F12 medium (pH 7.4) supplemented with 10% fetal bovine serum, 30 μg/ml endothelial cell growth supplement, and 10 μg/ml gentamycin.

### Parasites

*P. falciparum* FCR3, RP8 (able to bind CSA), and PAC2 (able to bind CD36 and ICAM-1) parasites were cultured on human 0^+^ erythrocytes in candle jars as described [13]. They were grown under standard culture conditions, replacing the 10% v/v human serum with 0.25% w/v Albumax (Life Technology, Paris, France). PRBC preparations were enriched to 80%–85% by gelatin flotation with Plasmion (Fresenius Kabi France, Couvier, France) [14], and suspensions were adjusted to 5 × 10^6^ PRBC/ml for cytoadherence assays.

### Inhibition of HBEC ICAM-1 and VCAM-1 Up-Regulation by LMP-420 upon TNF and LT Activation

HBEC-5i confluent monolayers were left unstimulated with and without treatment with LMP-420 (50 nM), or were activated with TNF (overnight or 6 h, 10 ng/ml) or with LT (overnight, 30 ng/ml), concomitantly or not with LMP-420 (50 nM), before analysis. HBEC-5i were then harvested and labeled by indirect labeling using mouse anti-human CD54 (ICAM-1 [84H10]) and CD106 (VCAM-1 [1G1]) antibodies (Beckman-Coulter Immunotech, Marseille, France), CD40 monoclonal antibody (mAb) (B-B20, Diaclone, Besançon, France), and CD36 mAb (FA6–152, gift from L. Edelman, Institut Pasteur Paris) as the first step. Secondary goat anti-mouse Alexa488–coupled mAb (Molecular Probes, Eugene, Oregon, United States) was added as the second step. A nonspecific isotype-matched mouse IgG1 (Beckman-Coulter Immunotech) was used for all controls. Cells were then resuspended in PBS before flow cytometry analysis on a Coulter Epics XL (Coultronics France, Margency, France). The area corresponding to HBEC-5i was defined, and mean fluorescence intensities of the positive cell populations were measured for each antigen.

### Effect of LMP-420 on Several Parasite Strains Cytoadherence to Activated HBEC

For cytoadherence assays, HBEC-5i were plated on 1% w/v gelatin-coated 12-well IFA slides and allowed to reach confluence. These cells were incubated for 18 h prior to the experiment, in the presence or absence of TNF (50 ng/ml) or LT (overnight, 30 ng/ml), concomitantly or not with LMP-420 (50 nM). EC were then washed with RPMI medium (pH 6.8) and incubated for 90 min at 37 °C with the parasite strains (ratio PRBC:EC was 50:1). Slides were then washed to remove non-adherent PRBC, and the remaining cells were fixed by incubation in 2.5% glutaraldehyde for 30 min, and bound RPBC were quantitated by microscopic analysis.

### Effect of LMP-420 on HBEC Vesiculation upon TNF or LT Stimulation

HBEC-5i were seeded and grown to confluence in 12-well culture plates. Cells were then left unstimulated or activated by TNF (100 ng/ml) or LT (100 ng/ml), concomitantly or not with LMP-420 (50nM), for 6 h. Culture supernatants were then collected and centrifuged at 1500 × *g* for 15 min to discard dead EC and debris. Endothelial microparticles were labeled using annexin V-FITC and then resuspended in binding buffer (Beckman-Coulter Immunotech) as previously described [15].

### Statistical Analysis

Statistical analyses were performed with GraphPad Prism version 4.0 for Windows, GraphPad Software (San Diego, California, United States. Data were analyzed by the Kruskall-Wallis and Dunn's pairwise tests. Results in the text are expressed as medians, interquartile ranges (IQR), and ranges of individual experimental groups. A value of *p* < 0.05 was considered significant.

## Results

We first investigated the inhibitory activity of LMP-420, the structure of which is illustrated in Figure 1, on HBEC-5i activation, i.e., cell adhesion molecule up-regulation upon TNF or LT stimulation. Among the major molecules present at the surface of EC (detailed in S. C. Wassmer, V. Combes, F. J. Candal, I. Juhan-Vague, and G. E. Grau, unpublished data), HBEC-5i in resting conditions were found to express constitutive cell adhesion molecules such as ICAM-1 and VCAM-1, and both were markedly up-regulated by TNF (Figure 2). In contrast, upon LT stimulation, only ICAM-1 was significantly up-regulated. The expression of CD40 was weakly induced by both TNF and LT, and CD36 was not detected on the surface of either resting or stimulated HBEC-5i. When added to resting cells, LMP-420 did not modify the expression of the surface molecules studied here. However, when LMP-420 was added 2 h before or simultaneously with TNF or LT, up-regulations observed for ICAM-1 and/or VCAM-1 with both stimuli were dramatically inhibited (Dunn's test, *p* < 0.01 in each condition), and the weak induction of CD40 by the two cytokines was abrogated.

**Figure 1:**
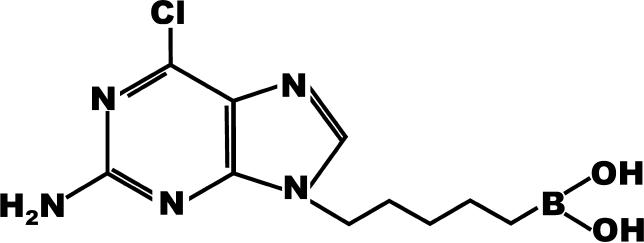
Structure of LMP-420 (2-NH_2_-6-Cl-9-[(5-Dihydroxyboryl)-Pentyl] Purine)

**Figure 2:**
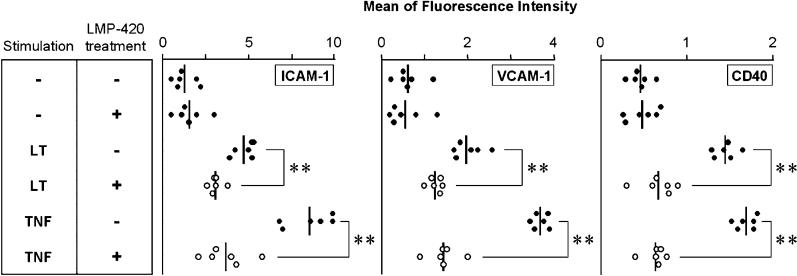
Inhibitory Effect of LMP-420 on TNF-Induced Up-Regulation of ICAM-1 and VCAM-1 HBEC-5i were left unstimulated or were activated with LMP-420 alone, TNF alone, TNF with LMP-420, LT alone, or LT with LMP-420. After an overnight incubation, cells were detached and analyzed by flow cytometry after immunolabeling for ICAM-1, VCAM-1, and CD40. Results are expressed as dotplots of mean fluorescence intensity of surface expression of the antigens measured in six experiments (Dunn's test, ** corresponds to *p* < 0.01).

We then investigated the consequences of such an inhibition on parasite cytoadherence to cerebral endothelium. To this end, we used PRBC infected with the FCR3 strain of *P. falciparum,* and subpopulations derived from it, selected for their adhesion to specific receptors. Thus, the RP8 subpopulation bound only to chondroitin sulfate A (CSA, which was found to be constitutively expressed by HBEC-5i), and the PAC2 subpopulation was selected for its capacity to adhere to ICAM-1 and CD36 [16]. HBEC-5i were cultured and left unstimulated or stimulated with TNF or LT, concomitantly or not with LMP-420, as described. After addition of PRBC, the slides were washed, and bound PRBC were counted on five randomly selected fields (0.28 mm^2^). Although the binding of CSA-specific-PRBC to resting or TNF-stimulated EC was constant in each condition, the level of ICAM-1/CD36-specific-PRBC binding was significantly higher in the presence of TNF as well as when cells were stimulated by LT (Dunn's test, *p* < 0.01; Figure 3A and 3B). However, under both conditions in the presence of LMP-420, this PAC2-PRBC binding increase was abrogated (Dunn's test, *p* < 0.01 for both TNF and LT; Figure 3A and 3B), and no statistical differences were observed between the cytoadherence in resting condition and upon LT- or TNF-stimulation in the presence of LMP-420 (*p* = 1 and *p* = 0.067, respectively). Even if there is no evidence of any differences in these experiments, it does not preclude the possibility that there are residual effects of TNF and/or LT in the presence of LMP-420. The FCR3 strain, composed of both CSA- and ICAM-1/CD36-specific subpopulations, exhibited a higher basal binding level (median: 705 PRBC/mm^2^; IQR: 110; range: 730.3 to 770.6 PRBC/mm^2^) than those of these two subclones taken separately (median: 448.5 PRBC/mm^2^; IQR: 79.2; range: 330.9 to 538.6 PRBC/mm^2^; and median: 239 PRBC/mm^2^; IQR: 74.25; range: 150.0 to 310.5 PRBC/mm^2^, respectively). The FCR3 strain's cytoadherence was increased upon both TNF and LT stimulation, but was significantly reduced in the concomitant presence of the cytokines and LMP-420 (Dunn's test, *p* < 0.01 for both TNF and LT; Figure 3A and 3B), and once again, no statistical differences were observed between the cytoadherence in resting condition and upon LT- or TNF-stimulation in the presence of LMP-420 (*p* = 0.4 and *p* = 0.294, respectively).

**Figure 3:**
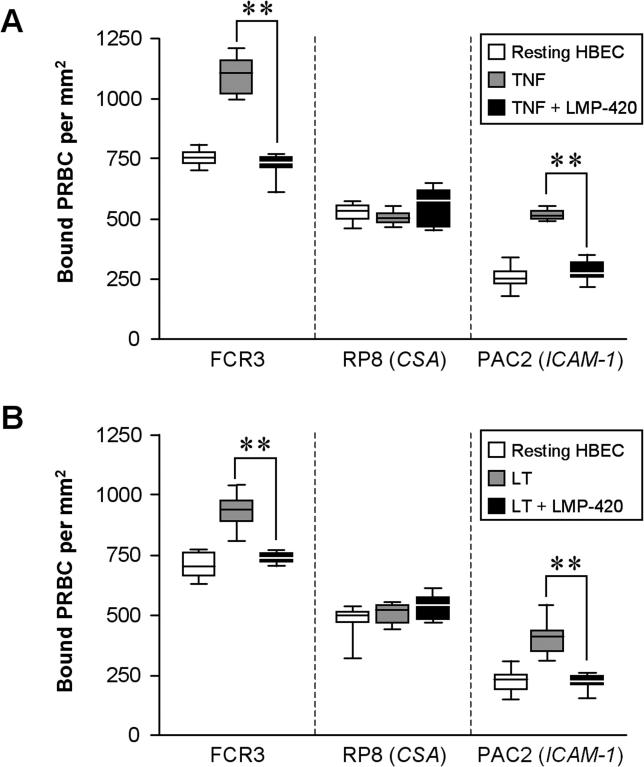
LMP-420 Inhibits TNF- and LT-Induced Increase of PRBC Cytoadherence HBEC-5i were cultured until confluence was reached and were left resting or activated as described earlier. Cytoadherence assays were then performed with FCR3-*P. falciparum* strain, and RP8 and PAC2 subpopulations selected for their ability to bind only CSA and ICAM-1/CD36, respectively (A and B). Results of six determinations in five experiments are expressed in bound PRBC, after two washing steps after the assay (Dunn's test, ** corresponds to *p* < 0.01).

To assess the blebbing and the shedding of MP from the HBEC-5i surface upon cytokine stimulation, cells were stimulated with TNF or LT, simultaneously with or without LMP-420. We quantitated the inhibitory effect of LMP-420 on activated HBEC-5i vesiculation by flow cytometry analysis. Upon both LT and TNF stimulation, HBEC-5i were shown to produce significantly higher MP numbers than in resting conditions (Dunn's test, *p* < 0.001). In the presence of LMP-420, the MP release was shown to be significantly reduced for both conditions of stimulation (Dunn's test, p < 0.001), and no differences were observed between TNF and LT (Figure 4).

**Figure 4:**
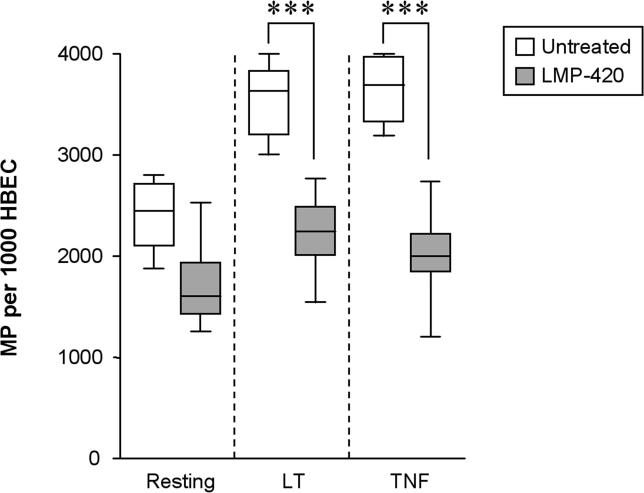
Inhibitory Effect of LMP-420 on LT- and TNF-Induced Increase of MP Release HBEC-5i were cultured and left resting or stimulated with TNF, TNF with LMP-420, LT, or LT with LMP-420 for 6 h before analysis. MP production was quantified by flow cytometry for each condition. Results (four determinations in four experiments) are expressed in numbers of MP labeled with annexin V-FITC, extracted from culture supernatants of 10^3^ HBEC-5i (Dunn's test, *** corresponds to *p* < 0.001).

## Discussion

Our results demonstrate that LMP-420, a newly discovered anti-inflammatory molecule, potently reduces endothelial activation, endothelial adhesiveness for *P. falciparum*-PRBC, and endothelial MP release, three major features of CM. To this end, we used a recently described in vitro CM lesion model based on the human brain microvascular EC line, HBEC-5i.

Our studies analyzed the effect of concomitant addition of LMP-420 and TNF or LT upon HBEC-5i phenotype modification. TNF and LT belong to the same family and interact with a common receptor, and it has been demonstrated that both cytokines may up-regulate the expression of the adhesion surface molecules ICAM-1 and VCAM-1 (S. C. Wassmer, V. Combes, F. J. Candal, I. Juhan-Vague, and G. E. Grau, unpublished data). In the presence of LMP-420 however, these up-regulations were markedly inhibited. Because LMP-420 has been shown to inhibit the release of both TNF and MCP-1 from LPS-activated PBMC, its effect on LT has not been shown so far. LMP-420 is a particularly potent inhibitor of TNF production, inhibiting up to 98% (S. Haraguchi, N. K. Day, W. Kamchaisatian, M. Engele, S. Stenger, N. Tangsinmankong, J. W. Sleasman, S. V. Pizzo, and G. J. Cianciolo, unpublished data).

We have investigated the effect of such inhibition of endothelial adhesion molecule up-regulation in the presence of pro-inflammatory cytokines on PRBC cytoadherence. After TNF or LT stimulations, we performed cytoadherence assays on HBEC-5i, with several subpopulations of *P. falciparum* FCR3 strains selected for their receptor specificities. The presence of LMP-420 concomitantly with TNF led to an abrogation of both the FCR3 and ICAM-1/CD36-specific PRBC cytoadherence increase observed when HBEC-5i were stimulated with TNF alone. This abrogation might be due to the inhibition of ICAM-1 up-regulation on EC surface, resulting from the addition of TNF and LMP-420, as described above. The cytoadherence of the CSA-specific PRBC subpopulation was not affected when HBEC-5i were previously activated with TNF or with TNF and LMP-420. These results are consistent with our previous data, demonstrating that HBEC-5i exhibit a stable and constant expression of CSA, which is not affected by a prior stimulation by TNF (S. C. Wassmer, V. Combes, F. J. Candal, I. Juhan-Vague, and G. E. Grau , unpublished data). Because the FCR3 parasite strain is a mixture of both previously used subpopulations [16], its basal binding level was higher than for CSA-specific or ICAM-1/CD36-specific PRBC subpopulations taken separately. However, its increased cytoadherence upon TNF-stimulation and its inhibition in the concomitant presence of TNF and LMP-420 probably reflects the ICAM-1-mediated cytoadherence of the ICAM-1/CD36-specific PRBC subpopulation. Indeed, because CD36 is lacking on HBEC-5i membrane and ICAM-1 expression dropped in the presence of TNF and LMP-420, the number of binding sites might be dramatically reduced. This could explain the ICAM-1/CD36-specific PRBC subpopulation cytoadherence decrease, leading to a FCR3 binding level similar to the level observed with the CSA-specific PRBC subpopulation. Indeed, CSA expression is not affected by TNF or LT, and its specific parasite cytoadherence was constant in both conditions. Similar abrogation of increased binding was observed for both FCR3 and ICAM-1/CD36-specific PRBC subpopulations when HBEC-5i were stimulated by LT in the presence of LMP-420 versus LT alone. These results suggest a dual specificity of the LMP-420 molecule, which can act on two of the majors cytokines involved in CM pathogenesis [10], leading in vitro to a reduction of the PRBC cytoadhesion of *P. falciparum* populations binding to ICAM-1. Because the degree of binding of these ICAM-1-specific PRBC was found to be highest in CM patients [17], we hypothesize that decreasing the cytoadhesion of these PRBC to endothelium in vivo might be beneficial.

Finally, we analyzed the effect of LMP-420, added simultaneously with TNF or LT, on endothelial vesiculation, a recently described marker of severity in pediatric CM patients. Upon both TNF and LT activation, HBEC-5i showed an increase of the MP release, and LMP-420 addition led to a significant, but partial, reduction of the vesiculation for both stimulation conditions. This vesiculation increase has been shown to be strongly associated with a fatal outcome in Malawian children who died of CM [5], and although its cause still remains unclear, the in vivo abrogation of MP release from activated endothelium may have a protective effect. Our data suggest that anti-inflammatory compounds, such as LMP-420, might provide a new way to inhibit this vesiculation, whether induced by TNF or LT.

LMP-420, a low molecular weight purine-based compound, exhibits anti-inflammatory activity that may be useful in targeting the wide variety of diseases in which TNF and its related family members play a role. TNF is a pleiotropic cytokine playing key roles in inflammation, resistance to infection, and cancer. Besides its beneficial actions, the pathogenic effects of TNF are evident in a wide variety of conditions, including CM. Several molecules inhibiting TNF, such as monoclonal antibody [18] or pentoxyfilline [19], have been tested in clinical trials, but failed to improve significantly the disease outcome. This could be explain by the fact that LT was recently demonstrated to also play a crucial role in the pathogenesis of this cerebral syndrome [9,10]. The data herein provide evidence for a dual inhibitory effect of LMP-420 on both TNF and LT in an in vitro model of CM lesion, when added before or simultaneously with both cytokines. Moreover, in the current context, efforts are being made to identify mechanisms regulating cytokine production, especially at the mRNA stability level [20,21]. LMP-420 is a molecule able to inhibit transcription of mRNA for TNF and LT in a variety of human cell types including monocytes, T lymphocytes, neutrophils, adipocytes, and EC, by interfering with responses involving the RelA (p65) component of NF-κB (G. Cianciolo, unpublished data).

In terms of potential side effects of anti-TNF therapies, LMP-420 has the advantage of the pharmacological control provided by a small molecule. The biological half-life of LMP-420 (based on studies in mice) is approximately 12 h. Thus, treatment could be suspended if clinical parameters worsened. The currently marketed TNF antagonists are designed to “bind and neutralize” all circulating TNF, which may also hinder immune responsiveness to some degree. Dose-response curves on LPS-activated human peripheral blood leukocytes indicate that even at 500 nM and 5000 nM (10-fold and 100-fold the IC_50_ dose, respectively), LMP-420 inhibits only 93% and 98% of the released TNF. Thus, the fact that a small amount of TNF may still be produced may actually have more of a physiological effect and allow the immune system to respond to the infectious agent by blunting the pathological concentrations of TNF that occur. We believe this aspect is extremely important, as developed earlier [22].

The findings presented here provide evidence that LMP-420 might be considered as a new therapeutic way for CM. Indeed, although there are currently three marketed TNF antagonist products (etanercept [Enbrel], a soluble TNF receptor [23]; infliximab [Remicade] humanized anti-TNF monoclonal antibody [24]; and adalimumab,[Humira] fully human anti-TNF monoclonal antibody [25]), none of them were described to present inhibitory effects on LT, and all three are large proteins. In addition to their being very costly, these molecules are less likely to cross the blood-brain barrier, whereas a small molecule like LMP-420 might have greater accessibility to the brain than protein-based therapeutics. Moreover, because of their proteinaceous nature, they require refrigerated shipping and storage. This is not the case for LMP-420, which is a small organic molecule, highly temperature stable. Finally, LMP-420 is of the same general class of compounds (purine nucleoside analogs) that has been safely used in humans for several decades, and thus could represent a novel, stable, and efficient therapeutic way to improve the outcome of CM patients. Although we recognize that experimental results observed in cell culture in vitro are not necessarily predictive of potential efficacy in either animal models or man, we believe that the results presented herein support the further evaluation of therapeutic strategies aimed at minimizing the pathologic host responses to Plasmodia-infected erythrocytes. Although it is entirely possible that the inflammatory process may be irreversibly advanced by the time a patient presents for treatment, there is currently insufficient information to know at what clinical stage intervention loses it value or could even become harmful. The availability of potent, small molecular weight cytokine inhibitors will hopefully allow us to determine that information.

**Patient Summary**

**Background**

The most severe complication of malaria is when it involves the brain, so-called cerebral malaria, and which is caused by one particular form of the malarial parasite, *Plasmodium falciparum*. Cerebral malaria occurs most frequently in children and is often fatal. Although it is not clear exactly what all the events in cerebral malaria are, it is known that there is inflammation, that some of the cells in the blood vessels become stimulated, and that red cells infected with malarial parasites adhere to the stimulated cells and get stuck in the small blood vessels of the brain.

**Why Was This Study Done?**

The researchers had previously identified a compound, LMP-420, that could block the synthesis of one of the substances that is associated with blood vessel stimulation. They wanted to test its effect in a model of cerebral malaria.

**What Did the Researchers Do and Find?**

They added LMP-420 to cultures of blood vessel cells in the presence or absence of the substances known to stimulate the blood vessel cells, as well as to a culture of red cells infected with the malarial parasite. They found that LMP-420 could prevent the activation of the blood vessel cells and could partially stop the infected red cells from sticking to the blood vessel cells. However, these results were observed only when LMP-420 was added either before or at the same time that the substances associated with blood vessel stimulation were present.

**What Do These Findings Mean?**

LMP-420 may be able to act as an anti-inflammatory substance in the blood vessels of the brain of patients infected with malaria. One drawback, however, is that the data thus far show efficacy only if given very early; in practice this would mean treating patients before they had symptoms of cerebral malaria, which might be practically difficult. However, there is no way to know from cell culture studies whether intervention after the process has started might benefit the final outcome. So, it will be necessary to test the compound further before it is clear whether it will be clinically useful.

**Where Can I Get More Information Online?**

Medline plus has many links to information about malaria: http://www.nlm.nih.gov/medlineplus/malaria.html

The CDC has a page on regional risks of malaria: http://www.cdc.gov/travel/regionalmalaria/

## Abbreviations

CM: cerebral malaria
CSA: chondroitin sulfate A
EC: endothelial cell
HBEC-5i: human brain–derived endothelial cells
IC_50_: 50% inhibitory concentration
IQR: interquartile range
LT: lymphotoxin α
mAb: monoclonal antibody
MP: microparticles
PRBC: parasitized red blood cells
TNF: tumor necrosis factor
